# Neuroprotective Effect of Bexarotene in the SOD1^G93A^ Mouse Model of Amyotrophic Lateral Sclerosis

**DOI:** 10.3389/fncel.2015.00250

**Published:** 2015-07-01

**Authors:** Javier Riancho, María Ruiz-Soto, María T. Berciano, José Berciano, Miguel Lafarga

**Affiliations:** ^1^Neurology Service, Centro de Investigación Biomédica en Red de Enfermedades Neurodegenerativas (CIBERNED), Instituto de Investigación Marqués de Valdecilla (IDIVAL), University Hospital Marqués de Valdecilla, University of Cantabria, Santander, Spain; ^2^Department of Anatomy and Cell Biology, Centro de Investigación Biomédica en Red de Enfermedades Neurodegenerativas (CIBERNED), Instituto de Investigación Marqués de Valdecilla (IDIVAL), University of Cantabria, Santander, Spain

**Keywords:** amyotrophic lateral sclerosis, bexarotene, G93ASOD1, mouse, proteostasis, retinoid-X receptor

## Abstract

Amyotrophic lateral sclerosis (ALS) is a neurodegenerative disease characterized by progressive weakness and muscle atrophy related to the loss of upper and lower motor neurons (MNs) without a curative treatment. There is experimental evidence suggesting that retinoids may be involved in ALS pathogenesis. Bexarotene (Bxt) is a retinoid-X receptor agonist used in the treatment of cutaneous lymphoma with a favorable safety profile whose effects have been recently investigated in other neurodegenerative diseases. In this study, we analyze the potential therapeutic effect of Bxt in the SOD1^G93A^ mouse model of ALS. Mice were treated with Bxt or vehicle five times per week from day 60 onward. Survival, weight, and neuromuscular function studies together with histological and biochemical analyses were performed. Bxt significantly delayed motor function deterioration, ameliorated the loss of body weight, and extended mice survival up to 30% of the symptomatic period. Histological analyses of the lumbosacral spinal cord revealed that Bxt markedly delayed the early motor-neuron degeneration occurring at presymptomatic stages in ALS-transgenic mice. Bxt treatment contributed to preserve the MN homeostasis in the SOD1^G93A^ mice. Particularly, it reduced the neuronal loss and the chromatolytic response, induced nucleolar hypertrophy, decreased the formation of ubiquitylated inclusions, and modulated the lysosomal response. As an agonist of the retinoic-X receptor (RXR) pathway, Bxt notably increased the nuclear expression of the RXRα throughout transcriptionally active euchromatin domains. Bxt also contributed to protect the MN environment by reducing reactive astrogliosis and preserving perisomatic synapsis. Overall, these neuroprotective effects suggest that treatment with Bxt could be useful in ALS, particularly in those cases related to SOD1 mutations.

## Introduction

Amyotrophic lateral sclerosis (ALS) is the most common disease affecting the motor neurons (MNs) with an annual incidence that ranges from 2 to 4 cases per 100,000 people. It is characterized by progressive loss of upper and lower MNs, accompanied by neuromuscular junction denervation. Death occurs within 2–5 years after diagnosis, usually due to respiratory failure (Kunst, 2004). Although several therapies have been tried, riluzole (a glutamate antagonist) is the only drug currently approved by the U.S. Food and Drug Administration (FDA) for ALS treatment. However, it has shown a very poor effect in patients’ survival (Cheah et al., 2010; Miller et al., 2012).

Approximately 90% of ALS cases are sporadic (sALS), whereas the remaining 10% have a hereditary origin and tend to cluster into families (fALS). Up to 20% of fALS cases are due to mutations of the gene encoding superoxide dismutase 1 (SOD1) (Pasinelli and Brown, 2006; Rothstein, 2009; Ferraiuolo et al., 2011). This is also the basis of a commonly used transgenic mouse model expressing the human *SOD1* gene with the G93A mutation (Gurney et al., 1994). High-copy SOD1^G93A^ transgenic mice recapitulate much of the pathophysiology of human ALS, including progressive MN degeneration, functional impairment, and reduced lifespan (Gurney et al., 1994). Furthermore, a relationship between SOD1 mutations and sALS has recently been reported supporting the translational value of studies using transgenic SOD1 mice (Bosco et al., 2010).

The mechanisms involved in ALS pathogenesis remain unclear and are thought to be multiple (Robberecht and Philips, 2013). Disturbances of RNA processing, oxidative and endoplasmic reticulum (ER) stress, impaired protein degradation with protein aggregation, axonal transport defects, and glial cell dysfunction represent some of the mechanisms that have been related to ALS (Corona et al., 2007; Schmidt et al., 2009; Lemmens et al., 2010; Andersen and Al Chalabi, 2011; Philips and Robberecht, 2011; Bendotti et al., 2012; Majounie et al., 2012; Hetz and Mollereau, 2014).

Biochemical analyses have also identified some pathways potentially involved in ALS pathogenesis, including retinoid pathways. Thus, it has been reported that wild type mice fed with a retinoid-free diet develop a clinical phenotype resembling ALS with loss of MNs in the anterior horn and forelimb paralysis (Corcoran et al., 2002). Additionally, Kolarcik and Bowser (2012) have suggested that retinoid signaling is altered in ALS. These authors showed that the pre-treatment of primary MN-enriched cultures with adapalene, a retinoic acid receptor beta (RARβ) agonist, protects MNs from cell death induced by oxidative stress. Furthermore, some reports propose that retinoic acid (RA) could modulate both the ubiquitin proteasome system (UPS) and the autophagy-lysosome pathways helping to maintain intracellular proteostasis (Rajawat et al., 2010; Anguiano et al., 2013; Cheng et al., 2013). Overall, these studies suggest a potential beneficial effect of retinoid pathway activation on neuron survival in MN-related diseases.

Bexarotene (Bxt) is a highly selective retinoid-X receptor (RXR) agonist with a favorable safety profile. As occurs with most of retinoids, hypothirodism, liver toxicity, and cutaneous disorders are the most common adverse reactions observed with its use (Duvic et al., 2001a,b). Bxt has been approved by the FDA for the treatment of cutaneous T-cell lymphoma, and is currently used as a long-term therapy in the clinical practice requiring periodic blood test monitoring (Farol and Hymes, 2004). This drug has already been tested in some mice models of neurodegenerative diseases. Thus, it has been shown that Bxt reduces the amyloid load in an Alzheimer disease mouse model and consequently induced a marked amelioration in the cognitive, social, and olfactory deficits (Cramer et al., 2012). However, other investigators have only partially reproduced these results (Fitz et al., 2013; Price et al., 2013; Veeraraghavalu et al., 2013). On the other hand, Bxt has also been tested in a mouse model of Parkinson’s disease exhibiting beneficial effects (McFarland et al., 2013). Therefore, based on the potential neuroprotective effects of retinoid pathway activation (Mey and Rombach, 1999; Schrage et al., 2006; Kolarcik and Bowser, 2012), we hypothesized that Bxt could be useful in ALS. We planned this study to investigate its effects on both motor function and MN degeneration and survival in the transgenic SOD1^G93A^ mouse model of ALS. Furthermore, we analyzed some potential neuroprotective mechanisms of Bxt including (i) cellular structures involved in proteostasis, such as protein synthesis machinery, nucleolus, and lysosomes, (ii) nuclear expression of the transcription factor RXRα, and (iii) reactive astroglial MN environment.

## Materials and Methods

### Animals and drug

Transgenic mice (B6SJLTg [SOD1-G93A] 1Gur/J) were obtained from The Jackson Laboratory (Bar Harbor, ME, USA) and maintained at the Animal Service of the University of Cantabria. The colony was maintained by mating heterozygous transgenic males with B6SJLF1/J hybrid females. Real time quantitative PCR (rtqPCR) of DNA obtained from tail tissue was used for genotyping, with specific primers detecting human SOD1 and the housekeeping mouse gene ApoB. Primer sequences were SOD1:GGG AAG CTG TTG TCC CAA G and CAA GGG GAG GTA AAA GAG AGC; ApoB: TCA CCA GTC ATT TCT GCC TTT G and GGG AAG CTG TTG TCC CAA G. Transgenic mice and control littermates were housed under controlled temperature and humidity, with a 12-h-light/12-h-dark cycle and free access to water and food. From day 105 of life, both transgenic and control mice were fed with Nutragel^®^ (Bio-serv Frenchtown, NJ, USA). The experimental protocol was approved by the Ethics Committee of the University of Cantabria following the Spanish legislation. Animals were equally distributed into the experimental groups taking into account littermate origin, sex, weight, neuromuscular score baseline values, and transgenic load.

Micronized Bxt (Targretin^®^, Eisai, Japan) was water dissolved (30 mg/ml) and administered to transgenic SOD1^G93A^ mice 5 days/week at a dose of 100 mg/kg/day by gastric gavage. Another group of mice were treated with vehicle (water) using the same protocol that Bxt-treated mice. The experimental groups included in the study are summarized in Table 1.

**Table 1:** **Experimental design**.

	Experimental group	*n*	Treatment	Start date (days)	End date (days)
**SURVIVAL AND NEUROMUSCULAR FUNCTION**					
Group 1	SOD1 G93A	15 (9m; 6f)	Bexarotene	60	Sacrifice
Group 2	SOD1 G93A	12 (7m; 5f)	Vehicle	60	Sacrifice
Group 3	Wild type	6 (3m; 3f)	Bexarotene	60	120
Group 4	Wild type	6 (3m; 3f)	Vehicle	60	120
**HISTOLOGICAL STUDIES**					
Group 5	SOD1 G93A	16 (10m; 6f)	Bexarotene	60	75/95
Group 6	SOD1 G93A	16 (10m; 6f)	Vehicle	60	75/95
Group 7	Wild type	12 (6m; 6f)	Vehicle	60	75/95
**BIOCHEMICAL STUDIES**					
Group 8	SOD1 G93A	10 (5m; 5f)	Bexarotene	60	95
Group 9	SOD1 G93A	10 (5m; 5f)	Vehicle	60	95
Group 10	Wild type	10 (5m; 5f)	Vehicle	60	95

### Survival, weight, and neuromuscular tests

To investigate long-term effects of Bxt administration, 27 transgenic mice were divided into two groups. Fifteen (9 males, 6 females) and 12 (7 males, 5 females) mice were treated with Bxt and vehicle, respectively. Additionally, 12 wild type mice were also divided into two groups and treated with Bxt or vehicle under the regimen previously described. Treatment begun on day 60 of life and continued until sacrifice. Animals were weighted three times per week beginning at day 60. After a 2-week training period, neuromuscular tests (Rotarod and hanging test) were recorded twice a week beginning at 9 weeks of age until the end of the experiment. A cylinder spinning at a constant speed of 12 rpm was used for the Rotarod test (Mouse Rota-Rod 47600, Ugo Basile, Argentina). On the other hand, a semi-spheric grid of 20 cm in diameter was used for the hanging test. The best of two attempts, which were separated by 10 min rest, was recorded. Following current recommendations (Vargas et al., 2013), animals were euthanized by the time they developed severe paralysis, when they were not able to straighten up after turning them on their back in the following 15 s.

### Histological analysis

To study histological changes, 2 groups of 16 transgenic mice each were treated with Bxt or vehicle from day 60 until day 95. Eight mice of each group were euthanized on days 75 and 95, respectively, and their spinal cords were processed for light or electron microscopy. The results were compared with those obtained in age-matched wild type mice.

### Light and confocal microscopy

After deep anesthesia with pentobarbital (50 mg/kg) mice were perfused with 3.7% paraformaldehyde in PBS (pH 7.4) for 15 min. The lumbosacral enlargement of the spinal cord (L2-S1) was dissected and post-fixed for 2 h. Spinal cord fragments were either dehydrated and embedded in diethylene glycol or processed for mechanical dissociation of MNs as previously reported (Pena et al., 2001).

For neuronal dissociation, small tissue fragments from the anterior horn were transferred to a drop of PBS on a siliconized slide. Then, a coverslip was applied on top of the slide and the tissue was squashed by percussion with a histologic needle in order to dissociate neuronal cell bodies. The preparation was then frozen in dry ice, and the coverslip removed using a razor blade. Using this procedure, most MNs remained adhered to the slide. Cell samples were processed in 96% ethanol at 4°C for 10 min, which increases the adhesion of cells to the slide, and rehydrated progressively in 70% ethanol and PBS. These squash preparations were used for propidium iodide (PI), a fluorescent staining of nucleic acids, and immunofluorescence.

Anterior horn cross sections, 2 μm thick, were processed for conventional hematoxylin–eosin staining, PI cytochemical staining, and immunofluorescence. The early loss of MN was evaluated at day 75 and 95 of life. The number of MNs was counted in four sections of each animal at segment L4 using hematoxylin–eosin staining. Only polygonal-shaped neurons present in ventral horns, larger than 20 μm and with prominent nucleoli, were used for the quantitative analysis as previously reported (Mancuso et al., 2014).

For immunofluorescence, tissue sections and squash preparations were sequentially treated with 0.5% Triton X-100 in PBS, 0.1M glycine in PBS containing 1% bovine serum albumin and incubated with the primary antibody overnight at 4°C. Then, the sections were incubated with the specific secondary antibody conjugated with FITC or Cy3 and mounted with Vectashield (Vector, USA). Confocal images were obtained with a LSM510 (Zeiss, Germany) laser scanning microscope using 40× oil or 63× oil (1.4 NA) objectives. In order to avoid overlapping signals, images were obtained by sequential excitation at 488 and 543 nm, to detect FITC and Cy3, respectively. Images were processed using Photoshop software.

To quantify astrogliosis, confocal images of the ventral horn’s gray matter immunostained for glial fibrillary acidic protein (GFAP) were recorded by using a 40× oil objective and the same confocal settings. Images were background corrected by reference regions outside the tissue and fluorescence intensities were estimated by using the ImageJ software (NIH, Bethesda, MD, USA; http://rsb.info.nih.gov/ij/).

The relative nuclear levels of RXRα in MNs of wild type, Bxt-treated and vehicle-treated transgenic SOD1^G93A^ mice were determined on 5 μm thick cryostat sections immunostained with the anti-RXRα antibody. Only anterior horn neurons larger than 20 μm and with prominent nucleoli were sampled. We used three animals per experimental group and at least 35 neurons per animal were sampled. Confocal images were recorded by using a 63× oil objective (NA 1.4) and the same confocal settings at a resolution of 1024 × 1024 pixels. The same immunostaining and image processing procedures were carried out for all the experimental groups. Images were background corrected by reference regions outside the tissue and fluorescence intensities of nuclear RXRα signal, excluding the nucleolus, were estimated by using the ImageJ software (NIH, Bethesda, MD, USA; http://rsb.info.nih.gov/ij/).

The following primary antibodies were used: goat polyclonal ­antibodies anti-choline acetyltransferase (ChAT) (Millipore, Billerica, MA, USA) and anti-cathepsin D (Santa Cruz Biotechnology, USA), and rabbit polyclonal antibodies anti-GFAP (Dako, Glostrup, Denmark), anti-SOD1 (Enzo Life Sciences, Switzerland), anti-ubiquitin-protein conjugates (Santa Cruz Biotechnology, USA), and anti-RXRα (D-20, Santa Cruz Biotechnology, USA).

### Electron microscopy

For electron microscopy, transgenic mice were perfused with 1% glutaraldehyde and 1% paraformaldehyde in 0.12M phosphate buffer. Tissue samples of the anterior horn were dehydrated and embedded in Araldite. Ultrathin sections were examined with a Jeol 201 electron microscope.

To determine the ultrastructural localization of cathepsin D in lysosomes, we performed immunogold electron microscopy. Tissue samples of the anterior horn fixed with 3.7% paraformaldehyde were dehydrated in methanol and embedded in Lowicryl K4M at −20°C. Ultrathin sections were sequentially incubated with 0.1M glycine in PBS (15 min), 5% BSA in PBS (30 min), and the anti-cathepsin D antibody diluted in PBS containing 0.1M glycine and 1% BSA (1 h at room temperature). After washing, the sections were incubated with an anti-goat secondary antibody conjugated to 10 nm gold-particles (BioCell, UK) diluted 1:25 in 1% BSA in PBS (45 min at room temperature). After washing, the sections were stained with uranyl acetate and lead citrate. Control sections were treated as described but omitting the primary antibody.

### Western blotting and real time quantitative PCR

Ten transgenic mice, treated with Bxt or vehicle from day 60 until day 95 of life, were used in these studies. For each biochemical assay (rtqPCR or WB) at least three mice of each animal group were used. Age-matched wild type mice receiving vehicle were used for comparison. After being anesthetized, they were decapitated and the lumbar spinal cord quickly removed and frozen in liquid nitrogen.

Gene expression was assessed by rtqPCR. RNA from spinal cord samples was isolated with Trizol following the manufacturer’s instructions (Invitrogen, Carlsbad) and purified with the RNeasy kit (Qiagen, Hilden, Germany) as previously reported (Delgado-Calle et al., 2011). Aliquots of RNA were reverse-transcribed with the PrimeScrip RT kit (Takara) using random hexamers as primers. Then, the expression of cathepsin D was determined rtqPCR using gene-specific primers and TaqMan probes (Life Technologies, Foster City, CA, USA). The threshold cycle (Ct) for each well was determined. The results were normalized to hypoxanthine phosphoribosyltransferase 1 (HPRT1). Relative gene expression was calculated as 2^−ΛCT^, where ΛCT is the difference between the gene of interest threshold cycle and the HPRT1 threshold cycle.

For WB analysis, the spinal cord samples (the ventral region of lumbosacral enlargement) were homogenized in a lysis buffer (100 mM NaCl, 10 mM Tris-HCl, pH 7.5, 1 mM EDTA, and 1 μg/ml aprotinin) and the homogenates were centrifuged. The protein in the supernatants were subjected to SDS-PAGE electrophoresis and afterwards transferred to polyvinylidene difluoride membranes, stained with antibodies, and visualized with the Odyssey system (LI-COR Biotechnology). Glyceraldehyde 3-phosphate dehydrogenase (GAPDH) and tubulin were used as protein loading controls. The antibodies used were a goat polyclonal anti-cathepsin D (Santa Cruz Biotechnology, Europe) and mouse monoclonal anti-GAPDH (Abcam, Cambridge, MA, USA). ImageJ software (U. S. National Institutes of Health, Bethesda, MD, USA) was used to quantify the density and size of the blots.

### Statistical analysis

The significance of the differences in weight was tested by unpaired Student’s *t*-test, while non-parametric Mann–Whitney *U* test was used to compare motor test scores of the Bxt- and vehicle-treated groups. The overall survival and time-course of motor deterioration plots were obtained with the Kaplan–Meier procedure, followed by between-group curve comparisons with the Gehan–Breslow–Wilcoxon test.

The number of MNs in the anterior horn was tested with ANOVA test. Intracellular ubiquitin aggregates, the relative nuclear levels of RXRα, and both the integrated GFAP and RXRα signal assessing astrogliosis were also tested with ANOVA test. The significance of differences of WB and rtqPCR was analyzed with non-parametric Kruskal–Wallis test. *p*-Values <0.05 were considered as significant. All analyses were two-tailed.

## Results

### Bxt treatment delays weight loss and extends survival in SOD1^G93A^ mice

**Figure 1:**
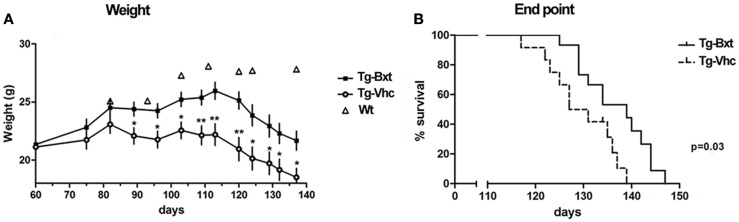
**Effects of bexarotene on weight and survival**. **(A)** Bexarotene treatment (Bxt) delays the start-up of weight loss as compared with the vehicle-treated (Vhc) transgenic SOD1^G93A^ mice. **(B)** Survival analysis showing increase of lifespan in the Bxt group as compared with the Vhc group. WT, wild type mice.

Bxt was well tolerated with no obvious signs of toxicity or drug-related deaths; in fact, the general condition was better preserved in the transgenic mice treated with Bxt than in vehicle-treated transgenic mice. Animals that received vehicle started to lose weight at day 80, while those receiving Bxt continued gaining weight until day 110. Bxt-treated mice weighed up to 20% more than controls. Differences in weight were significant from day 90 onward (Figure 1A). In comparison with wild type mice, the weight of transgenic mice was 75 and 89% in the vehicle-treated and Bxt-treated groups, respectively. Bxt-treated transgenic animals remained more physically active than controls receiving vehicle. However, Bxt administration to wild type mice induced no significant changes in either body weight or neuromuscular tests.

Bxt treatment also prolonged the survival of SOD1^G93A^ transgenic mice. The first death occurred on day 117 in the control group and on day 125 in the Bxt group. The median survival was 10 days longer in the group of animals treated with Bxt [139 vs. 129 days; Gehan–Breslow–Wilcoxon test, *p* = 0.03; hazard ratio = 0.29, 95% confidence intervals (CI): 0.11–0.80] (Figure 1B).

### Bxt preserves the neuromuscular function delaying the disease onset in SOD1^G93A^ mice

**Figure 2:**
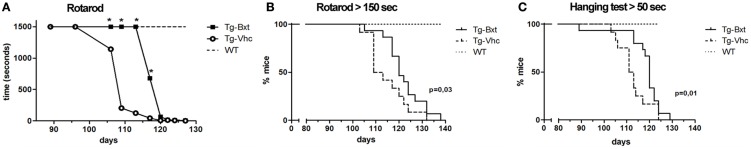
**Bexarotene treatment enhances motor performance in SOD1^G93A^ mice**. Bexarotene treatment (Bxt) delays neuromuscular function deterioration as assessed by Rotarod **(A,B)** and hanging **(C)** tests.

Disease onset assessed by neuromuscular tests was also significantly delayed in Bxt-treated mice. Wild type mice and transgenic mice prior to disease onset were consistently able to stand for more than 1500 s on the Rotarod spinning wheels. The Rotarod median performance in the vehicle-treated transgenic mice started to decrease on day 96, while in the Bxt-treated animals did not change till day 113 (Figure 2A). The median time to reach a threshold of marked deterioration (established at 150 s in the present study) was 110 days in the vehicle-treated group and 119 days in the Bxt-treated group (Gehan–Breslow–Wilcoxon test, *p* = 0.033; hazard ratio = 0.42; 95% CI: 0.16–1.07) (Figure 2B). Similarly, wild type mice and presymptomatic transgenic mice were consistently able to hang for more than 600 s. Deterioration of the four-limb hanging test scores appeared to begin in both transgenic mice groups approximately on day 100. However, vehicle-treated mice showed more rapid deterioration than Bxt-treated animals. From day 110 to day 124, Bxt-treated animals were able to cling for twice as much time in comparison to those receiving vehicle (*p*-values 0.03–0.05). Significant between-group differences also existed regarding the time to reach stages of moderate or advanced deterioration. Thus, the median age to hang <50 s was 112 days in vehicle-treated mice and 120 days in the Bxt-treated group (Gehan–Breslow–Wilcoxon test, *p* = 0.01; hazard ratio = 0.36; 95% CI: 0.13–0.96) (Figure 2C). Similarly, significant between-group differences existed when a 10-s score was considered (*p* = 0.006).

### Bxt partially preserves MN cytology and ameliorates the spinal MN loss in SOD1^G93A^ mice

**Figure 3:**
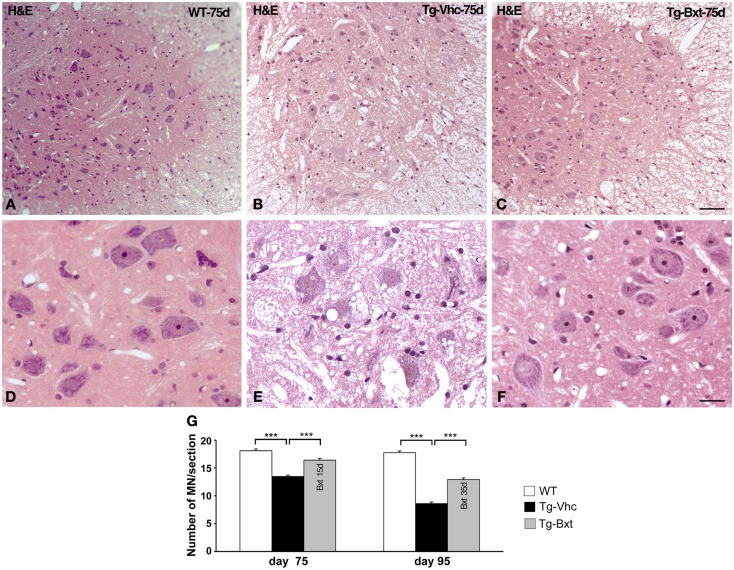
**Light microscopy findings in the anterior horn**. **(A–C)** Representative examples of anterior horn cross sections stained with hematoxylin–eosin from 75 day-old wild type [WT **(A)**] mice and vehicle-treated [Tg-Vhc **(B)**] and Bxt-treated [Tg-Bxt **(C)**] transgenic SOD1 ^G93A^ mice. The gray matter in the WT mouse shows the typical organization of MNs and a compact neuropile. Note that while MNs appear well preserved in the Tg-Bxt image, the Tg-Vhc image exhibits a reduced number of MNs and a spongiform alteration of the neuropile. Scale bar: 50 μm. **(D–F)** Higher magnification images illustrate the prominent vacuolar degeneration of MNs in the Tg-Vhc transgenic mice. Scale bar: 25 μm. **(F)** Quantitative analyses of spinal cord MNs at 75 and 95 days of age **(G)** (***p < 0.001).

As expected, the ongoing expression of the mutant transgene induced the development of neuromuscular changes in both groups of transgenic mice. However, our results showed that chronic administration of Bxt delayed the appearance of clinical manifestations of the disease. Therefore, we decided to explore whether the drug also delay histological alterations at presymptomatic stages. At day 75, marked abnormalities were observed in transgenic mice receiving vehicle, by contrast no gross abnormalities were observed in their behavior or motor performance. By conventional hematoxylin–eosin staining, the anterior horn of control wild type mice showed the typical organization of MNs with prominent nucleoli and Nissl bodies in addition to a compact neuropile (Figures 3A,D). Conversely, degenerative signs were observed in the anterior horn of vehicle-treated SOD1^G93A^ mice. They consisted of cytoplasmic vacuolization of some MNs and spongiform changes of the neuropile (Figures 3B,E). These alterations were much less pronounced in transgenic mice treated with Bxt, where both MNs and neuropile generally appeared well preserved (Figures 3C,F).

We next analyzed the MN loss at both presymptomatic and early-symptomatic stages. This was assessed by evaluating the number of MN cell bodies in cryosections of the anterior horn (segments L4 and L5) stained with PI that fulfilled the previously referred criteria (Mancuso et al., 2014). On day 75, vehicle-treated SOD1^G93A^ mice had 13.5 ± 0.23 MNs per section, while Bxt-treated SOD1 mice had 16.4 ± 0.25 MNs per section (74 and 90%, respectively, of MNs in wild type mice) (Figure 3G). The differences were more marked, at day 95 of life, when in the vehicle-treated group there were 8.6 ± 0.3 MNs per section, whereas 12.9 ± 0.30 MNs were present in Bxt-treated mice (48 and 72%, respectively, of MNs in wild type mice). Differences were significant at both stages (*p* < 0.001) (Figure 3G).

**Figure 4:**
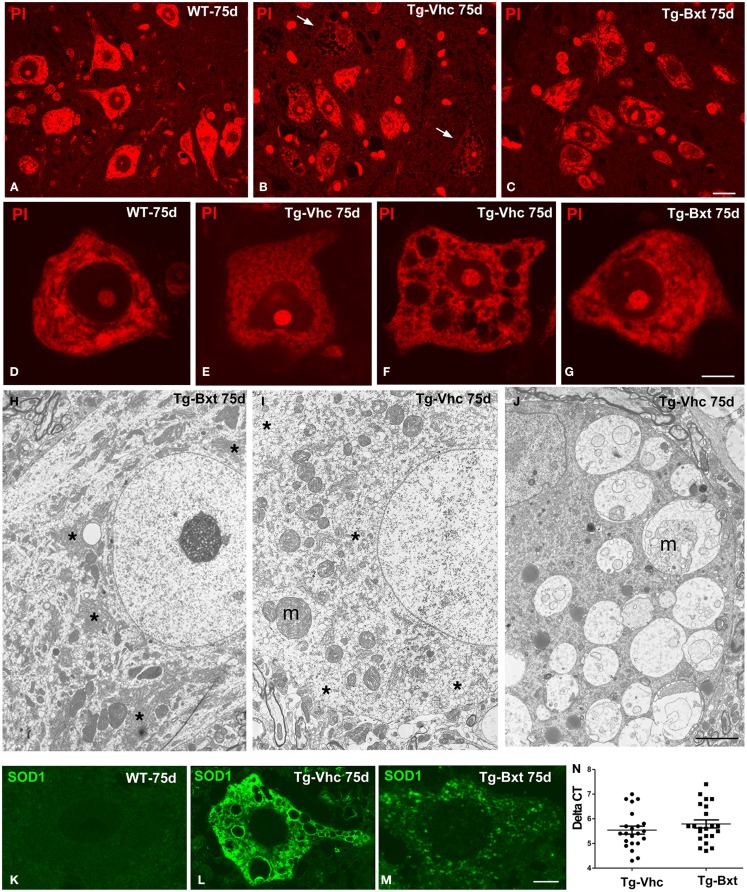
**Morphological alterations of MNs**. Representative examples of anterior horn cross cryosections stained with PI from 75 days old wild type [WT **(A)**] mice and vehicle-treated [Tg-Vhc **(B)**] and Bxt-treated [Tg-Bxt **(C)**] transgenic SOD1^G93A^ mice. Note that the prominent nucleoli in all MNs and the well-preserved Nissl bodies in both WT and Tg-Bxt animals. Several MNs in the anterior horn of the Tg-Vhc mouse exhibit a severe cytoplasmic disruption with vacuolar degeneration and chromatolysis (white arrows). Scale bar: 20 μm. **(D–G)** Higher magnification images of dissociated MNs stained with PI from WT **(D)**, Tg-Vhc **(E–F)**, and Tg-Bxt mice **(G)**. Note that the typical distribution of Nissl bodies in the WT MN **(D)** and different stages of chromatolysis and vacuolar degeneration in MNs from the Tg-Vhc mouse **(E,F)**. Scale bar: 7.5 μm. **(H–J)** Electron micrographs of MNs from Tg-Bxt **(H)** and Tg-Vhc transgenic SOD1^G93A^ mice **(I,J)**. The MN from the Tg-Bxt mouse exhibits a well preserved nuclear and cytoplasmic structure, including typical arrays of RER cisterns (asterisks). MNs from the Tg-Vhc mice show severe chromatolysis (asterisks), extensive cytoplasmic vesiculation, mitochondrial abnormalities (m), and accumulation of large vacuoles derived from altered mitochondria (m). Scale bars: **(H)**, 4 μm; **(I,J)**, 3 μm. **(K–M)** Representative confocal microscopy images of MNs immunolabeled for SOD1 from the WT **(K)** and Tg-Vhc **(L)** and Tg-Bxt **(M)** SOD1^G93A^ mice. Strong immunostaining signal of SOD1 appears throughout the cytoplasm, excluding the lumen of large vacuoles, in the MN of the Tg-Vhc mouse. Scale bar: 5 μm. **(N)** Transgene expression in Bxt-treated and Vhc-treated transgenic mice.

The morphological changes observed by conventional hematoxylin–eosin staining were further characterized by PI staining and ultrastructural analysis. On day 75, fluorescent staining of nucleic acid with PI revealed prominent nucleoli and Nissl bodies in healthy wild type MNs (Figures 4A,D) and different levels of chromatolysis in MNs of the vehicle-treated transgenic SOD1^G93A^ mice (Figures 4B,E,F). Large cytoplasmic vacuoles unstained with PI were commonly observed in advanced stages of MN degeneration (Figure 4F). In contrast, most MNs in Bxt-treated SOD1^G93A^ mice exhibited well-preserved Nissl bodies and prominent nucleoli (Figures 4C,G). Electron microscopy analysis on day 75 confirmed that the preservation of the basic cellular organization in MNs of Bxt-treated SOD1^G93A^ mice. This included the normal structure of the nucleolus and chromatin as well as the cytoplasmic domains enriched in rough endoplasmic reticulum (RER) and free polyribosomes (Figure 4H). In contrast, a severe disruption of RER (chromatolysis), associated with the presence of abnormal and swelling mitochondria and different degrees of vacuolar degeneration, was frequently observed in MNs of the vehicle-treated SOD1^G93A^ mice (Figures 4I–J).

The pathogenic basis of the transgenic SOD1^G93A^ mouse model lies on the over-expression of the toxic SOD1^G93A^ protein (Jaarsma et al., 2000; Sau et al., 2007; Brotherton et al., 2012). To confirm the validity of the experimental model, we decided to investigate if chronic administration of Bxt modulated the expression of the transgene. Interestingly, rtqPCR analysis revealed no significant differences in human SOD1 mRNA levels between Bxt-treated and vehicle-treated transgenic mice (Figure 4N). Additionally, we also assessed the mutant SOD1 protein distribution by immunofluorescence. While MNs from wild type mice showed a weak diffuse staining of the endogenous SOD1 (Figure 4K), a strong fluorescent signal corresponding to the mutant SOD1 was clearly detected in the cytoplasm of MNs from SOD1^G93A^ mice. Moreover, SOD1 immunoreactivity was absent from the nucleus (Figures 4L,M). At day 75, accumulations of mutant SOD1 were commonly observed throughout the cytoplasm, excluding vacuoles, in degenerating MNs from vehicle-treated transgenic mice (Figure 4L). However, a lower cytoplasmic concentration of this mutant protein, visible as a diffuse staining and small aggregates, was found in MNs of Bxt-treated mice (Figure 4M). These findings suggest that Bxt partially prevents mutant protein aggregation and preserves neuronal proteostasis until more advanced stages.

**Figure 5:**
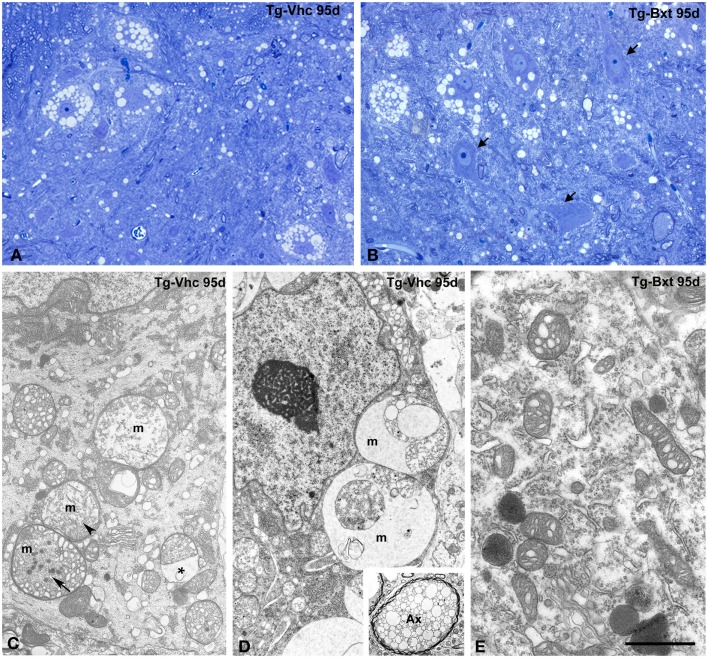
**Morphological alterations of MNs at symptomatic stages of neurodegeneration**. **(A,B)** Toluidine blue staining of gray matter sections from vehicle-treated [Tg-Vhc **(A)**] and Bxt-treated [Tg-Bxt **(B)**] transgenic SOD1^G93A^ mice at day 95. The **(A)** shows MN loss and prominent vacuolar degeneration of remnant cell bodies. The **(B)** illustrates well-preserved neuronal bodies (arrows) coexisting with other ones exhibiting different levels of vacuolar degeneration. Scale bar: 20 μm. **(C–E)** Representative electron micrographs of MNs from Tg-Vhc **(C,D)** and Tg-Bxt **(E)** mice at advanced stages of neurodegeneration. **(C,D)** The cell bodies show large vacuoles containing remnants of degenerated mitochondria (m), with dilatation of the intermembranous space (asterisk) and presence of dense granules (arrows), in addition to cytoplasmic accumulation of small vesicles and nuclear eccentricity. The inset illustrates a typical axonal vacuolar degeneration of a myelinated fiber (Ax). **(E)** Detail of cytoplasm from a Bxt-treated MN illustrating the initial dilation of mitochondrial cristae. Scale bars: **(C–E)** 3 μm, inset 2.6 μm.

The histological analysis of the anterior horn at symptomatic stages of disease (95 days old) shows morphological alterations in both groups of transgenic SOD1^G93A^ mice. Thus, light microscopy analysis of toluidine blue stained semithin sections clearly demonstrated the presence of degenerating MNs (Figures 5A,B). However, whereas most MNs exhibited severe vacuolar degeneration in vehicle-treated mice (Figure 5A), some well-preserved MNs coexisted with others that displayed different levels of vacuolar degeneration in Bxt-treated mice (Figure 5B). In both transgenic groups of animals, the ultrastructural analysis of MNs in advanced stages of neurodegeneration revealed (i) mitochondrial alterations, including dilation of cristae, presence of dense granules in the matrix, and global swelling resulting in the formation of large vacuoles, (ii) disruption of the RER, (iii) presence of lipid droplets, (iv) proliferation of neurofilaments, and (v) frequently nuclear eccentricity (Figures 5C–E). Moreover, axonal degeneration of myelinated fibers was a common finding in the neuropile at symptomatic stages (Figure 5D, inset). As expected, histological analysis of animals euthanized at the full paralysis stage showed similar abnormalities in both groups.

### Bxt induces nucleolar hypertrophy in spinal cord MNs of SOD1^G93A^ mice

**Figure 6:**
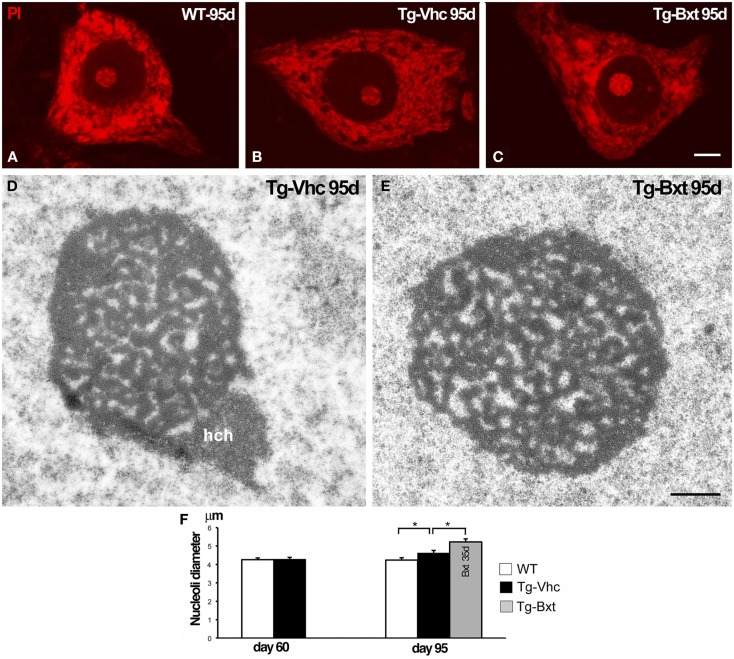
**Nucleolar response of MNs in transgenic SOD1^G93A^ mice**. Higher magnification images of dissociated MNs stained with PI from wild type [WT **(A)**], vehicle-treated [Tg-Vhc **(B)**], and Bxt-treated [Tg-Bxt **(C)**] mice at day 95. MNs from Bxt-treated mice have a larger nucleolus. Scale bar: 5 μm. **(D,E)** Representative electron micrographs of MN nucleoli from [Tg-Vhc **(D)**] and [Tg-Bxt **(E)**] illustrating their reticulated configuration (hch: heterochromatin mass). Scale bar **(D,E)**: 1 μm. **(F)** Morphometric analysis of nucleolar diameters of MNs at 60 and 95 days old (*p < 0.05).

It is well established that the nucleolus plays an essential role in the synthesis of rRNAs and molecular assembly of pre-ribosomal particles (Raska et al., 2006; Boisvert et al., 2007). Indeed, the nucleolar configuration and size correlated with the transcriptional activity of the ribosomal genes in mammalian neurons (Kinderman and Jones, 1993; Berciano et al., 2007; Hetman and Pietrzak, 2012; Palanca et al., 2014). To determine the possible contribution of the nucleolus to the neuroprotective effects of Bxt, we have analyzed the structural organization and size of MN nucleoli. Squash preparations of MNs stained with PI revealed the euchromatic nuclear configuration and the presence of prominent nucleoli with a high intensity of rRNA signal in wild type mice as well as in both vehicle-treated and Bxt-treated transgenic mice (Figures 6A–C). Morphometric analysis of nucleolar diameter on presymptomatic day 60 (before starting Bxt treatment) did not reveal significant differences between wild type and transgenic SOD1^G93A^ mice. However, at an early-symptomatic stage (95 days old), the nucleolar diameter of MNs was significantly higher in MNs of SOD1^G93A^ mice treated with Bxt for 30 days than those in MNs of both wild type and vehicle-treated SOD1^G93A^ mice (Figure 6F). Electron microscopy analysis confirmed this nucleolar hypertrophy, and also revealed the reticulated nucleolar configuration, with numerous fibrillar centers, in most of MNs of both vehicle-treated and Bxt-treated SOD1^G93A^ mice (Figures 6D,E). The Bxt-induced increase in nucleolar size is consistent with a neuroprotectetive response of the nucleolus for enhancing ribosome biogenesis.

### Bxt contributes to modulate cellular proteostasis in the spinal cord MNs of SOD1^G93A^ mice

**Figure 7:**
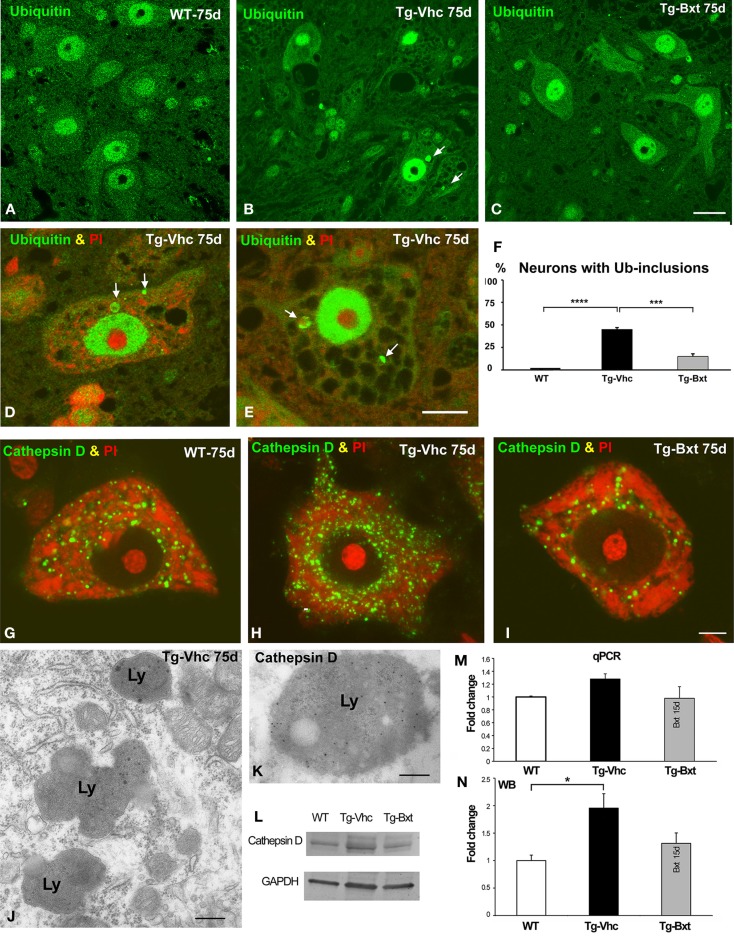
**Alterations of proteostasis in MNs: the UPS and lysosome pathway**. **(A–C)** Representative confocal images of anterior horn semithin sections immunostained for ubiquitin-protein conjugate from wild type mice [WT **(A)**], vehicle-treated [Tg-Vhc **(B)**], and Bxt-treated [Tg-Bxt **(C)**] transgenic SOD1^G93A^ mice. Note that the presence of ubiquitin-positive cytoplasmic inclusions (white arrows) in MNs from the Tg-Vhc mouse **(B)**. Scale bar: 25 μm. **(D,E)** Detail of MNs from Tg-Vhc mice immunostained for ubiquitin and counterstained with PI. The MN in **(D)** shows the presence of ubiquitin-positive cytoplasmic inclusion (arrows) and small Nissl bodies. A prominent vacuolar degeneration and ubiquitin-positive inclusions (arrows) are observed in the MN of **(E)**. Scale bar **(D,E)**: 10 μm. **(F)** Quantitative analyses of the proportion of MNs containing ubiquitylated inclusions (****p < 0.0001; ***p < 0.001). **(G–I)** Representative examples of dissociated MNs immunostained for cathepsin D and counterstained with PI from WT **(G)**, Tg-Vhc **(H)**, and Tg-Bxt SOD1^G93A^ mice **(I)**. The MN of the Tg-Vhc mouse shows greater accumulation of lysosomes **(H)** than those observed in both WT and Tg-Bxt **(G,I)**. Scale bar **(G–I)**: 5 μm. **(J)** Electron micrograph of the cytoplasm of a MN from Tg-Vhc mouse illustrating the accumulation of lysosomes. Scale bar: 750 nm. **(K)** Immunogold electron microscopy image illustrating the localization of the cathepsin D in the electron-dense lysosomal matrix. Scale bar: 500 nm. **(L–N)** Western blot and real time rtqPCR analysis of cathepsin D (*p < 0.05).

Protein aggregation is a common feature of many neurodegenerative diseases (Ross and Poirier, 2004). The presence of ubiquitylated protein aggregates has been widely described in ALS mice, consistent with a dysfunction of cellular proteostasis (Robberecht and Philips, 2013). This prompted us to investigate whether Bxt reduces protein aggregation into ubiquitylated cytoplasmic inclusions. Immunostaining of semithin sections of the anterior horn with an antibody that recognizes ubiquitin-protein conjugates revealed a high-nuclear concentration of ubiquitylated proteins, excluding the nucleolus, and a diffuse cytoplasmic signal in MNs from both wild type and transgenic SOD1^G93A^ mice (Figures 7A–C). Ubiquitin-positive cytoplasmic inclusions were commonly found in MNs exhibiting different levels of vacuolar degeneration and chromatolysis in vehicle-treated SOD1^G93A^ mice (Figures 7B,D,E). The quantitative analysis of the proportion of MNs containing ubiquitin-positive cytoplasmic inclusions (Figure 7F) showed a significant reduction in Bxt-treated SOD1^G93A^ mice as compared with vehicle-treated mice (14% vs. 43%, *p* < 0.001). These findings strongly support a Bxt-induced enhancement of proteostasis resulting in lower levels of protein aggregation.

Together with the UPS, the autophagy-lysosome pathway is the other major proteolytic system (Korac et al., 2013). To evaluate the lysosomal response in transgenic SOD1^G93A^ mice, we investigated the expression pattern of the protease cathepsin D, as a molecular marker of lysosomes. Immunostaining for cathepsin D in squash preparations counterstained with PI revealed the abundance of cathepsin D-positive lysosomes interspersed between Nissl bodies in wild type MNs (Figure 7G). However, a notable greater accumulation of lysosomes, particularly at the perinuclear cytoplasm, was clearly visible in vehicle-treated transgenic mice (Figure 7H). Interestingly, most MNs in Bxt-treated transgenic mice displayed a cathepsin D pattern similar to that observed in wild type mice (Figure 7I). Conventional electron microscopy analysis showed the accumulation of lysosomes in MNs of vehicle-treated transgenic mice (Figure 7J). Their lysosomal nature was confirmed with immunogold electron microscopy for cathepsin D. Gold particles decorated the cathepsin D-rich electron-dense matrix while the lipid-rich electron-lucent areas were free of immunolabeling (Figure 7K). Biochemical analyses with rtqPCR and WB demonstrated an increase in cathepsin D mRNA and protein levels in the anterior horn of vehicle-treated transgenic SOD1^G93A^ mice as compared with both Bxt-treated SOD1^G93A^ and wild type mice (Figures 7L–N).

### Bxt treatment increases nuclear expression levels of RXRα in MNs

**Figure 8:**
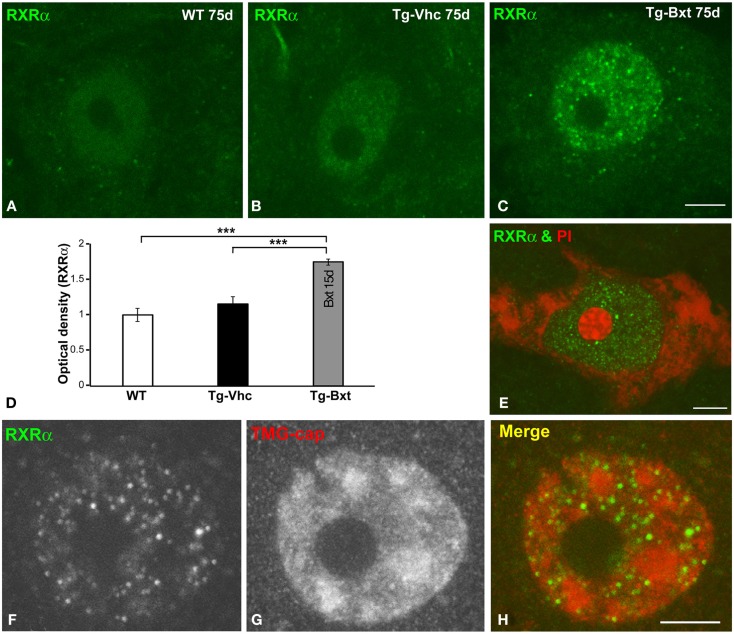
**Nuclear expression of the RXRα in MNs**. **(A–C)** Immunofluorescence for RXRα in MN nuclei from wild type [WT **(A)**], vehicle-treated [Tg-Vhc **(B)**], and Bxt-treated [Tg-Bxt **(C)**] transgenic SOD1^G93A^ mice. **(D)** Densitometric analysis of nuclear fluorescence intensity of RXRα signal in MNs (*p* < 0.001). **(E)** Double labeling for RXRα and PI in a MN from Bxt-treated SOD1^G93A^ mouse revealed the absence of RXRα signal in the nucleolus. **(F–H)** Co-immunostaining for RXRα and TMG-cap in a MN nucleus from Bxt-treated SOD1^G93A^ mouse. Note that the distribution of RXRα microfoci in euchromatin domains and their absence within the nuclear speckles. Scale bars: 4 μm.

On the basis that Bxt is an agonist of nuclear RXR receptors, which are ligand-dependent transcription factors, we investigated whether the neuroprotective effect of Bxt treatment could be partially mediated by an increase in the nuclear expression of RXRα in MNs. At day 75, confocal microscopy images of immunolabeled MNs revealed a weak diffuse nuclear pattern of RXRα expression, excluding the negative nucleolus, in wild type mice (Figure 8A). A similar pattern but with the presence of some microfoci of low RXRα immunoreactivity was found in MNs of transgenic SOD1^G93A^ mice (Figure 8B). Interestingly, Bxt-treatment induced a remarkable increase in nuclear RXRα immunolabeling with a non-homogeneous distribution. Thus, RXRα appeared concentrated in numerous microfoci with higher fluorescence intensities than those observed in vehicle-treated MNs. These bright microfoci stood out over the diffuse nuclear staining (Figure 8C). In addition to the negative nucleolus, several irregular areas with very low or no RXRα signal were observed throughout de nucleus (Figure 8C). The measurement of nuclear (excluding nucleolus) fluorescent intensity of RXRα on confocal images, using the ImageJ software, showed a significant increase of this nuclear receptor signal in Bxt-treated MNs as compared with both wild type and vehicle-treated transgenic SOD1^G93A^ mice (Figure 8D).

To further characterize the nuclear compartmentalization of RXRα in Bxt-treated neurons, we performed double labeling experiments combining RXRα detection with PI, as a marker of the neuronal nucleolus (Palanca et al., 2014), demonstrating the absence of RXRα in this nuclear compartment (Figure 8E). Moreover, we performed co-immunostaining for RXRα and the TMG-cap of spliceosomal small nuclear ribonucleoproteins (snRNPs), as a marker of nuclear speckles of splicing factors. These nuclear structures are DNA free nuclear domains involved in the storage and processing of pre-mRNA splicing factors (Lamond and Spector, 2003). Interestingly, nuclear microfoci of RXRα appeared distributed in euchromatin regions and were conspicuously absent from the DNA free nuclear speckles immunolabeled with the anti-TMG-cap antibody (Figures 8F–H). However, RXRα microfoci were frequently observed at the periphery of nuclear speckles (Figure 8H).

### Bxt contributes to preserve the MN environment in spinal cord of SOD1^G93A^ mice

**Figure 9:**
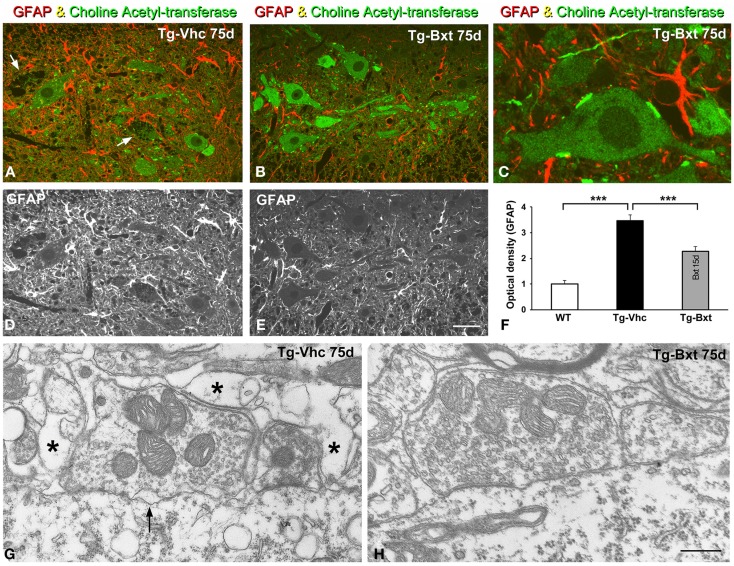
**Reactive astrogliosis and synaptic alterations**. **(A–E)** Representative confocal images of anterior horn sections coimmunolabeled for ChAT and GFAP from Vhc-treated [Tg-Vhc **(A,D)**] and Bxt-treated [Tg-Bxt **(B,C,E)**] transgenic SOD1^G93A^ mice. Note that the prominent reactive astrogliosis and vacuolar degeneration of MNs (white arrows) in the Tg-Vhc mouse **(A,D)**. Scale bar: **(A,B,D,E)**, 30 μm; **(C)**, 10 μm. **(F)** Densitometric analysis of fluorescence intensity of GFAP (***p < 0.001). **(G,H)** Electron micrographs of perisomatic synapses with their associated perisynaptic glia from Tg-Vhc **(G)** and Tg-Bxt **(H)** MNs. Note that the well preserved synaptic structure in the MN from the Tg-Bxt mouse **(H)** and the disruption of the synaptic cleft (arrow) and perisynaptic glia (asterisks) in the MN from the Tg-Vhc mouse **(G)**. Scale bar: 1 μm.

It is well accepted that astrocytes are involved in ALS pathogenesis in both human patients (Haidet-Phillips et al., 2011) and rodent models (Yamanaka et al., 2008). To determine the potential neuroprotective effects of Bxt treatment on the reactive astroglial response to MN neurodegeneration, we performed GFAP immunostaining on semithin (2 μm thick) sections of the anterior horn. As illustrated in Figures 9A–E, reactive astrogliosis with hypertrophic astrocytes, which is a common feature of SOD1^G93A^ mice (Hall et al., 1998), was less pronounced in the anterior horn of Bxt-treated mice. Thus, at day 75, the densitometric analysis of GFAP fluorescence intensity measured in the gray matter of vehicle-treated and Bxt-treated SOD1^G93A^ mice increased by 3.5 and 2.3 times, respectively, respect to wild type mice (*p* < 0.001, wt vs. both Tg-Vhc and Tg-Bxt; *p* < 0.001, Tg-Vhc vs. Tg-Bxt) (Figure 9F). These findings reflect a Bxt-induced down-regulation of the reactive astroglial response.

Another important point of the astroglial response is the reorganization of the perisynaptic glia that enwraps synapses, excluding the synaptic cleft, and modulates synaptic transmission (Reichenbach et al., 2010). Cholinergic synaptic boutons on MN cell bodies and dendrites were immunolabeled with the anti-ChAT antibody. Double immunostaining for GFAP and ChAT revealed the well-preserved morphology of the ChAT-positive MNs and their synaptic boutons in Bxt-treated SOD1^G93A^ mice (Figures 9B,C). In contrast, the vacuolar degeneration and the loss of ChAT-positive synapses were prominent features in MNs from vehicle-treated SOD1^G93A^ mice (Figure 9A). By day 75, electron microscopy analysis of perisomatic synapses confirmed the well preserved structure of both synaptic boutons and perisynaptic glia in Bxt-treated mice (Figure 9H). Conversely, severe structural alterations, namely dilatation of the synaptic cleft, detachment of synapses, and severe disruption of the perisynaptic glia, were found in the majority of MNs of mice treated with vehicle (Figure 9G).

## Discussion

Amyotrophic lateral sclerosis is one of the most disabling and ominous neurodegenerative diseases, being Riluzole, with a modest effect on survival, the only drug that can be currently offered to patients. Therefore, there is a pressing need to find new therapies to cure or at least ameliorate the severe course of the disease. Our study was undertaken to contribute to the search of new drugs for ALS patients.

Our results indicate that Bxt has a beneficial effect in transgenic SOD1^G93A^ mice. In particular, treatment with this retinoid agonist significantly delayed the disease onset and extended lifespan. As expected, Bxt did not prevent the development of the disease, since it did not block the expression of the mutant transgene. This fact increases the external viability of our findings. Overall, our results suggest that Bxt exerts a neuroprotective effect by interacting with multiple cellular pathways. The main target of Bxt, the RXR, appeared to be overexpressed in MN nuclei of mice treated with the drug, confirming its target enhancement in the spinal cord after oral administration.

Some of early cellular alterations reported here and in previous studies (Chiu et al., 1995; Bendotti et al., 2001; Pasinelli et al., 2004; Zang et al., 2005; Martin et al., 2007; Koval et al., 2013; Vinsant et al., 2013a,b) in MNs of the SOD1^G93A^ mouse include vacuolar degeneration of cell bodies and neuronal processes, mitochondrial alterations, presence of intracellular protein aggregates, synapse disruption, progressive loss of MNs, and astrogliosis in the anterior horn. All these changes reveal a severe neuronal dysfunction preceding clinical manifestations. Our histological results are consistent with the notion that Bxt, although unable to block the main pathophysiological mechanism, since it did not modify the mutant transgene expression, delayed disease onset by preserving MN homeostasis until more advanced stages.

A main target of Bxt, the RXRα, appeared overexpressed in MN nuclei of mice treated with the drug, suggesting that the neuroprotective effect of Bxt was partially mediated by the activation of the RA signaling pathway. A similar neuronal nuclear localization of RXRα has been previously reported after spinal cord contusion injury, suggesting that the participation of the RA signaling in the physiological response to CNS injury (Schrage et al., 2006). When activated, RXR are known to promote transcription of numerous target genes (Evans and Mangelsdorf, 2014). Our results show a significant increase in the nuclear expression of RXRα in MNs of Bxt-treated SOD1^G93A^ mice in comparison with MNs of vehicle-treated SOD1^G93A^ mice, suggesting a Bxt-induced up-regulation of protein-coding target genes. In this vein, RXRα appears distributed throughout the extensive nuclear domains of euchromatin (active chromatin), which is a chromatin configuration characteristic of MNs (Riancho et al., 2014), but it is excluded from the nucleolus and DNA free nuclear speckles of splicing factors (Lamond and Spector, 2003). Moreover, the numerous nuclear microfoci of RXRα induced by Bxt treatment have an organization and spatial distribution similar to those of transcription factories identified in neurons using the *in situ* transcription assay with 5′-fluorouridine incorporation into nascent RNA (Casafont et al., 2006). Interestingly, some of these RXRα microfoci appear in the periphery of nuclear speckles, where specific highly active genes have been shown to localize (Xing et al., 1993; Moen et al., 2004). Taken together, the extensive nuclear localization of RXRα in MNs of Bxt-treated SOD1^G93A^ mice supports that this nuclear receptor is actively engaged in transcriptional activation of target genes and presumably in epigenetic changes via chromatin remodeling (Evans and Mangelsdorf, 2014). This interpretation is also consistent with the recent RXR cistrome data indicating the existence of many thousands of genomic binding sites for a single nuclear receptor affecting elaborate networks of genes (Tang et al., 2011). In fact, several reports have suggested that retinoids play a protective role in degenerative and acquired neurological diseases, such as Alzheimer or stroke (Sato et al., 2008; Crockett et al., 2011; Obulesu et al., 2011; Shimada et al., 2013).

Retinoids modulate the expression of hundreds of genes, including a large number of neuronal genes (Lane and Bailey, 2005; Evans and Mangelsdorf, 2014). In the case of ALS, retinoid-regulated neuronal genes might impact on important cellular processes, such as the antioxidant response (SOD1), neuroinflammation and immune modulation (VEGF, IL2, p65 subunit of NFkβ), cytoskeletal organization (neurofilament L, M, H proteins), ion transport (K^+^ channel Kir2.1, L-type and N-type Ca^++^ channels), intracelular signaling (phospholipase A2, CREB, PKA), and synaptic homeostasis (ChAT, vesicular ACh and GABA transporters, NMDA receptor NR1 and kainate receptor GluR6) (Lane and Bailey, 2005; Kolarcik and Bowser, 2012). Overall, these effects could be partially responsible of the neuroprotective effect of Bxt in the transgenic SOD1 mouse model of ALS. However, the precise mechanism of action of retinoids in ALS remains to be elucidated. Although in a preliminary study Crochemore et al. (2009) reported that chronic administration of all-*trans* RA had a negative effect in transgenic ALS mice by reducing lifespan, our results are consistent with several studies supporting the neuroprotective role of retinoids. First, the administration of a retinoid-free diet in rats induced a phenotype similar to ALS, indicating that a defect in retinoid signaling could promote MN disease (Corcoran et al., 2002). Second, gene expression profiling in both ALS animal models and sALS patients have demonstrated altered transcription of genes related to retinoid pathways (Malaspina et al., 2001; Jokic et al., 2007; Malaspina and Turkheimer, 2007). Third, it has also been reported that RA signaling facilitates axon outgrowth and nerve regeneration, whereas its disruption leads to MN degeneration (Maden, 2007). Moreover, Kolarcik and Bowser (2012) have recently analyzed the expression of genes of the retinoid pathway in the spinal cord of ALS patients and found that different isoforms of RAR correlated with the survival of MNs. In line with this, the authors demonstrated that pre-treatment of primary MNs-enriched cultures with a pan-RAR or RARβ-specific agonist decreased MN cell death associated with oxidative stress, while a RARβ-specific antagonist enhanced cell death. On the basis that RXR can also act as heterodimers with peroxisome proliferator-activated receptor (PPAR) and liver X receptor (LXR), future experimental studies combining treatments with PPAR/LXR agonists and Bxt could be considered. This combined therapy might potentially enhance the neuroprotective effect of Bxt on MN survival.

As in other neurodegenerative diseases, oxidative and ER stresses with disturbed proteostasis are considered relevant features in ALS (Rothstein, 2009; Parakh et al., 2013; An et al., 2014). Consistent with a proteostasis dysfunction, we have observed lysosomal proliferation, with up-regulation of cathepsin D expression and increased cytoplasmic aggregation of aberrant proteins into ubiquitylated inclusions in MNs of the vehicle-treated SOD1^G93A^ mice. Several studies correlate the disturbance of neuronal proteostasis with an impairment in the UPS and secondary dysfunction of autophagy-lysosome pathway (Martinez-Vicente and Cuervo, 2007). Although this pathway constitutes a physiological mechanism to degrade proteins that “escape” from UPS and is beneficial for cell welfare (Tan et al., 2014), dysfunction of lysosomal proteolysis can be extremely harmful for neuronal function and survival (Ghavami et al., 2014). Indeed, up-regulation of the neuronal lysosomal system with increased levels of capthesin D is a prominent finding in Alzheimer’s disease (Cataldo et al., 1995; Adamec et al., 2000). Similarly, altered mRNA and protein levels of cathepsin D have been correlated with more severe MN degeneration in a transgenic mouse model of ALS (Wootz et al., 2006). The up-regulation of the lysosomal system reported here, in the SOD1^G93A^ mouse, may simply reflect a compensatory response of MNs to the accumulation of aberrant proteins. However, several studies support a potential direct role of cathepsin D as mediator of neurotoxicity and cell death through abnormal permeabilization of lysosome and enzyme leakage into cytoplasm (Adamec et al., 2000; Boya and Kroemer, 2008).

As expected, when compared with wild type mice, our transgenic SOD1^G93A^ mice showed protein aggregates, identified as ubiquitylated inclusions, which are a pathological hallmark of ALS (Ferraiuolo et al., 2011; Bendotti et al., 2012). There is previous evidence that over-expression of mutant SOD1 protein tends to collapse the UPS by inducing ER stress, resulting in secondary accumulation of misfolded proteins and a severe disturbance of neuronal proteostasis (Saxena et al., 2009). The higher cytoplasmic accumulation of ubiquitylated inclusions and lysosomes in MNs from the vehicle-treated SOD1^G93A^ than in MNs from Bxt-treated SOD1^G93A^ mice suggests that Bxt treatment reduces proteostasis dysfunction. Consistent with this potential neuroprotective effect of Bxt on neuronal proteostasis, Rajawat et al. (2011) have recently reported that RA promotes the autophagy-lysosome pathway by inducing the acidification and maturation of the autophagosome through a mechanism independent of the classic retinoid nuclear receptors. Moreover, a recent study indicates that the administration of RA has a neuroprotective effect under conditions of UPS inhibition (Cheng et al., 2013). In line with this concept, our results suggest that MNs from Bxt-treated SOD1^G93A^ mice could have a greater ability to control proteostasis, and particularly to clear aberrant proteins, than MNs from vehicle-treated SOD1^G93A^ mice. Upon detection of a pathobiological signal, neurons commonly activate a neuroprotective compensatory response that, if detrimental conditions persist, precedes the activation of the neurodegeneration and cell death. In this context, our results support that Bxt treatment delays the intricate progression of neuroprotection to neurodegeneration in ALS spinal cord MNs.

Spinal cord MNs from both wild type and transgenic SOD1^G93A^ mice had prominent nucleoli with a reticular configuration (Riancho et al., 2014; present results). The nucleolar hypertrophy detected in MNs of Bxt-treated transgenic SOD1^G93A^ mice seems to reflect a reactive nucleolar response to enhance nucleolar transcription and ribosome biogenesis. It is well established that nucleolar size positively correlates with both cell size and the transcription rate of rDNA genes (Raska et al., 2006; Berciano et al., 2007; Palanca et al., 2014). Enlargement of nucleolar size with up-regulation of ribosomal (rRNA) gene expression has previously been reported in experimental models of neuronal injury, such as axotomy and treatment with proteasome inhibitors (Kinderman and Jones, 1993; Palanca et al., 2014). Nucleolar enlargement also occurs in MNs of ALS patients with very rapid clinical course (Wakayama, 1992). The higher nucleolar size of the MNs of Bxt-treated mice, as compared with those of vehicle-treated mice, suggests an enhanced Bxt-induced compensatory response of the nucleolus to the ER stress caused by the mutant SOD1 protein (Kikuchi et al., 2006; Saxena et al., 2009). This neuroprotective response could sustain ribosome biogenesis and translational activity in order to delay the disruption of the protein synthesis machinery (chromatolysis) in transgenic SOD1^G93A^ mice.

A remarkable finding here is the Bxt-induced reduction of astrogliosis combined with better preservation of the perisynaptic glia in transgenic SOD1^G93A^ mice. Both cellular events also support a neuroprotective response. Reactive astrogliosis with hypertrophy and hyperplasia of astrocytes is a well-known response to degeneration and neuron loss. In the case of ALS, astrogliosis has been reported in both patients and murine models of the disease (Hall et al., 1998; Fischer et al., 2004). The slowdown of the astrogliosis response in Bxt-treated mice is consistent with the minor neurodegenerative signs observed in MNs. The involvement of astrogliosis in ALS pathogenesis is also supported by a recent study showing that spinal cord astrocytes expressing ALS-linked mutant SOD1 release cytotoxic factors to MNs (Nagai et al., 2007). Regarding the possible cellular effects of Bxt on astrocyte behavior, a previous study in an experimental model of astrocyte–neuron co-culture demonstrated that treatment with all-*trans* RA induced the differentiation and attenuated the astrocyte proliferation (Wohl and Weiss, 1998). This finding suggests that retinoids can contribute to down-regulate the proliferation (hyperplasia) of astrocytes in the transgenic SOD1^G93A^mice.

Synaptic alterations and their associated excitotoxicity in MNs have been widely studied in ALS as pathogenic mechanisms (Van Den et al., 2006; Sunico et al., 2011; Casas et al., 2013). In addition to the ultrastructural alterations in synaptic boutons previously reported in the SOD1^G93A^ mice (Sunico et al., 2011), our results show a severe disruption of the perisynaptic glia that surround perisomatic MN synapses. Perisynaptic glia plays an important role in maintaining synaptic homeostasis through different functions, such as neurotransmitter traffic regulation, energy recycle, and ion homeostasis (Belanger and Magistretti, 2009; Reichenbach et al., 2010). The current findings support that these regulatory mechanisms may be overwhelmed in the SOD1^G93A^ mice, contributing to disease progression. In this vein, preservation or delayed disruption of perisynaptic glia in Bxt-treated SOD1^G93A^ mice could also represent an untapped potential mechanism of neuroprotection.

Several authors have reported a variety of treatments in the SOD1^G93A^ mouse model of ALS. In view of our results, Bxt seems to be slightly better than riluzole and similar to other promising drugs recently tested (Gurney et al., 1998; Mancuso et al., 2014). In the future, mixed approaches combining drugs with synergic therapeutic effects will be worthwhile. In our view, one advantage of Bxt is that is a drug currently used in the clinical practice, a fact that would definitely facilitate future therapeutic trials.

In conclusion, the results of the present study demonstrate that chronic treatment with Bxt delayed disease onset, preserved motor function and extended survival in transgenic SOD1^G93A^ mice. Multiple mechanisms may be involved in this neuroprotective effect, including the activation of the RXR target genes. Bxt ameliorated the MN loss in the ALS mice by preserving the organization of the cellular structures directly involved in proteostasis, such as the protein synthesis machinery and the lysosomes. The Bxt-induced amelioration of proteostasis disturbance was also supported by the decrease of ubiquitylated cytoplasmic inclusions and by the neuroprotective nucleolar response. Additionally, Bxt contributed to protect MN environment by reducing reactive astrogliosis and synaptic alterations. Although further studies are needed to fully elucidate the molecular mechanisms involved in neuroprotective effects of Bxt, this drug appears to be a promising neuroprotective therapy for ALS, especially for those cases related to SOD1 mutations.

## Conflict of Interest Statement

The authors declare that the research was conducted in the absence of any commercial or financial relationships that could be construed as a potential conflict of interest.
